# Autophagosome marker, LC3, is released extracellularly via several distinct pathways

**DOI:** 10.1002/2211-5463.70150

**Published:** 2025-11-10

**Authors:** Koki Saito, Masashi Arakawa, Koki Maeda, Eiji Morita

**Affiliations:** ^1^ Department of Biochemistry and Molecular Biology, Faculty of Agriculture and Life Science Hirosaki University Japan; ^2^ Division of Biomolecular Function, Bioresources Science, United Graduate School of Agricultural Sciences Iwate University Morioka Japan

**Keywords:** autophagic secretion, autophagy, HiBiT tag, knock‐in cells, LC3 family, lysosome

## Abstract

Autophagy‐mediated secretion contributes to the maintenance of intracellular homeostasis by releasing cytoplasmic components into the extracellular space. However, several aspects of the process remain unclear. In this study, we developed an ultrasensitive detection system using HiBiT tag/NanoBiT technology to analyze the conditions that trigger the secretion of LC3, an autophagosome marker. In HiBiT‐tagged knock‐in cells, a detectable amount of HiBiT‐dependent NanoLuc luciferase activity (HiBiT activity) from HiBiT‐fused LC3 was observed in the culture supernatants. However, the levels were lower than those of CD63. HiBiT activity was detected only in the presence of detergent, indicating that LC3 was released from the lipid membranes. Treatment with bafilomycin A1 significantly increased the extracellular HiBiT activity, which was diminished in ATG5 or FIP200 knockout cells, suggesting that this release depends on autophagosome formation. However, some HiBiT‐LC3 was detected in these knockout cells, indicating that LC3 may be released via an autophagy‐independent mechanism. The introduction of a C‐terminal truncation (ΔG) or the K51A/L53A mutation also reduced LC3 release, but did not completely inhibit it, suggesting that multiple pathways exist for LC3 release. This system is expected to elucidate the mechanisms underlying autophagy‐mediated secretion.

## Graphical abstract

This study establishes a novel HiBiT‐tagging system for ultrasensitive detection of LC3, revealing multiple pathways for its extracellular secretion. It demonstrates that LC3 is released via both autophagy‐dependent and ‐independent mechanisms, including a novel route for nonlipidated LC3‐I. This versatile tool provides unprecedented quantitative insights into autophagic secretion, fundamentally advancing our understanding of cellular homeostasis and its regulatory pathways.

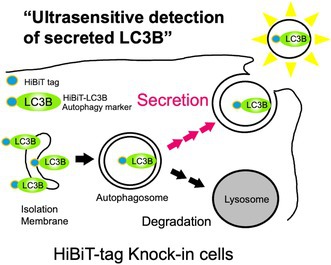

**Abbreviations**
AMPK: AMP‐activated protein kinase
ANOVA: analysis of variance
ATG: autophagy‐related gene/protein
BafA1: bafilomycin A1
Cas9: CRISPR‐associated protein 9
CRISPR: clustered regularly interspaced short palindromic repeats
DMEM: Dulbecco's modified Eagle medium
EV: extracellular vesicle
FBS: fetal bovine serum
FRAP: fluorescence recovery after photobleaching
GFP: green fluorescent protein
gRNA: guide RNA
HBSS: Hank's balanced salt solution
HEATR3: HEAT repeat containing 3
HiBiT: high‐affinity BiT (a small peptide tag for NanoBiT technology)
KI: knock‐in
KO: knockout
LAMP1: lysosomal‐associated membrane protein 1
LAP: LC3‐associated phagocytosis
LC3: microtubule‐associated protein 1A/1B‐light chain 3
LIR: LC3‐interacting region
mTOR: mammalian target of rapamycin
NanoBiT: NanoLuc binary technology
NanoLuc: a small, bright luciferase enzyme
NDP52: nuclear dot protein 52 kDa
OPTN: optineurin
PBS: phosphate‐buffered saline
PCR: polymerase chain reaction
PE: phosphatidylethanolamine
PEI: polyethylenimine
ppt: precipitate
SDS/PAGE: sodium dodecyl sulfate/polyacrylamide gel electrophoresis
SNARE: soluble *N*‐ethylmaleimide‐sensitive factor attachment protein receptor
STX: syntaxin
sup: supernatant
TFEB: transcription factor EB
TOMM20: translocase of outer mitochondrial membrane 20
VAMP: vesicle‐associated membrane protein
V‐ATPase: vacuolar‐type H^+^‐ATPase
WT: wild‐type

Autophagy is a bulk degradation system that transports cytoplasmic components to lysosomes and is conserved across all eukaryotic organisms, from budding yeast to humans [1, 2]. As the isolation membrane elongates and encloses its targets, an organelle called an autophagosome is formed. Autophagosomes then fuse with lysosomes, where the enclosed materials are degraded [3, 4, 5]. Although autophagy is activated under nutrient starvation, selective autophagy removes specific targets and plays a crucial role in the maintenance of cellular homeostasis [6, 7, 8].

LC3, a key component of autophagy in mammalian cells, is a homolog of yeast ATG8 [9, 10, 11]. Six types of LC3 have been identified in mammalian cells: LC3A, LC3B, LC3C, GABARAP, GABARAPL1, and GABARAPL2. After LC3 is translated as a precursor (proLC3), the 22 amino acids at the C terminus are cleaved by the cysteine protease ATG4 to generate LC3‐I [9, 11, 12]. As autophagy progresses, LC3‐I is conjugated to phosphatidylethanolamine (PE) through a system involving the enzymes ATG3, ATG5, ATG7, ATG10, ATG12, and ATG16L1, which results in the formation of LC3‐II [9, 11, 13]. LC3‐II is anchored to the autophagosome membrane and localized on both the inner and outer surfaces [9, 13]. LC3‐II on the outer membrane is cleaved by ATG4 and converted into LC3‐I. In contrast, the LC3‐II in the inner membrane is degraded when autophagosomes fuse with lysosomes [11, 14]. The ratio of LC3‐I to LC3‐II is correlated with the number of autophagosome membranes, making LC3‐II a reliable marker of autophagic activity [9]. Furthermore, in selective autophagy, substrate recognition is mediated by the direct interaction between LC3 on the isolation membrane and autophagy receptors such as p62, nuclear dot protein 52 kDa, and optineurin [15, 16]. Disruption of this molecular mechanism of intracellular homeostasis has been linked to the development of neurodegenerative diseases, cancer, bacterial infections, and inflammatory conditions [17, 18, 19, 20, 21].

In the recently identified autophagy secretion pathway, cytoplasmic components isolated by the autophagy machinery are transported and secreted extracellularly rather than delivered to lysosomes [22]. This secretory pathway is believed to be activated when intracellular degradation is impaired owing to lysosomal dysfunction [22, 23]. This may be a compensatory mechanism that helps maintain intracellular homeostasis by releasing cytoplasmic components or specific target molecules into the extracellular space [23, 24, 25, 26]. LC3 and autophagy receptors are primarily secreted within the small extracellular vesicle (EV) fraction (40–150 nm) rather than within the large EV fraction (150–1000 nm) encapsulated within membrane‐bound vesicles during secretion [22]. Furthermore, because defects in core ATG factors reduce autophagy secretion, autophagy‐dependent secretion may occur during intracellular degradation [22, 25, 26]. This pathway has also been implicated in the secretion of factors without signal peptides, such as tissue inhibitor of metalloproteinase‐1, interleukin‐1β, and ferritin [24, 25, 26]. However, many aspects of autophagy remain unknown, including whether autophagy occurs in normal cells, the precise conditions that trigger autophagy, and the extent to which different target substances are secreted.

This study developed an ultrasensitive detection system using HiBiT tag/NanoBiT technology to analyze the conditions that trigger LC3 secretion, measure the amount of LC3 secreted, and identify the pathways involved in its secretion. The results show that LC3 is secreted through different pathways under specific conditions, providing new insights into the largely unknown process of autophagy‐related secretion.

## Materials and methods

### Cell culture and reagents

The 293T cells were cultured at 37 °C in the presence of 5% CO_2_ in Dulbecco's modified Eagle's medium with high glucose (DMEM; Nacalai Tesque, Kyoto, Japan) containing 10% fetal bovine serum (FBS, lot number: 2404079; Gibco, Thermo Fisher Scientific Inc., Waltham, MA, USA), 50 units·mL^−1^ of penicillin (Nacalai Tesque), and 50 μg·mL^−1^ of streptomycin (Nacalai Tesque). Genome‐edited cells established in this study were cultured under the same conditions. The antibodies, chemicals, cells, and software used in this study are listed in Table 1.

**Table 1:** Resources table.

Antibodiis	Source	Identifier	Dilution factor	Experiment
Rabbit polyclonal anti‐FIP200	GeneTex	Cat#GTX129093	1:1000	WB
Mouse monoclonal anti‐ATG5	MBL	Clone:4D3	1:1000	WB
Mouse monoclonal anti‐HiBiT	Promega	clone 30E5	1:10000,1:1000	WB,IF
Rabbit polyclonal anti‐LC3	MBL	Cat#PM036	1:1000	WB
Rabbit polyclonal anti‐p62	MBL	Cat#PM045	1:1000	WB
Mouse monoclonal anti‐α‐Tubulin	Sigma‐Aldrich	Clone: DM1A	1:1000	WB
Mouse monoclonal Tom20	Santa Cruz Biotechnology	sc‐17764,Lot # H0320	1:1000	WB
Mouse monoclonal anti‐CD63	DSHB	H5C6	1:1000	WB
anti‐Mouse IgG	Jackson ImmunoResearch	115‐035‐003	1:3000	WB
anti‐Rabbit IgG	Jackson ImmunoResearch	111‐035‐003	1:3000	WB

Chemicals				
DMSO	nacalai tesque	Lot No. V2T2615		
HBSS(‐)	nacalai tesque	Lot No. L3T5204		
Bafilomycin A1	AdipoGen LIFE SCIENCES	Cat# BVT‐0252‐C100		
MG132	Enzo	BML‐PI102‐0005		
puromycin	InvivoGen	ant‐pr‐1		
Hoechest (33342)	Thermo Fisher Scientific	H1399	1:1000	IF

Reagent		
Nano Glo HiBiT Extracellular Detection System	Promega	Cat#N2421

Experimental models: Cell lines		
HEK293T	ATCC	Cat# CRL‐3216
HiBiT‐LC3B KI 293T	Established by this study	
HiBiT‐LC3B KI/ATG5 KO 293T	Established by this study	
HiBiT‐LC3B KI/FIP200 KO 293T	Established by this study	
HiBiT‐CD63 KI 293T	Maeda *et al*. [27]	
HiBiT‐Tomm20 KI 293T	Established by this study	

Software and algorithms		
CRISP‐ID	Dehairs *et al*. [28]	https://gbiomed.kuleuven.be/english/research/50488876/51819059/crisp‐id
CHOPCHOP	Labun *et al*. [29]	https://chopchop.cbu.uib.no/
GraphPad Prism 10	GraphPad Software Inc.	https://www.graphpad.com/
Fiji‐ImageJ	Schindelin *et al*. [30]	https://imagej.net/softwarw/fiji/

### Construction of plasmid DNA and DNA transfection

All plasmid DNAs used in this study were constructed using either ligation or Gibson assembly methods [31]. Information regarding all the plasmids used in the experiments is presented in Table 2. In the ligation method, genes amplified using polymerase chain reaction (PCR) are ligated into various restriction enzyme sites in the plasmid vector. In the Gibson assembly method, PCR was performed using primers designed to create > 15‐base homologous sequences at the ends of both the plasmid vector and the insert. The purified plasmid vector and insert were mixed with Gibson assembly master mix (New England Biolabs, Ipswich, MA, USA) and Dpn1 (New England Biolabs) and incubated at 37 °C for 30 min, followed by incubation for 60 min at 50 °C to assemble the DNA fragments. Plasmid transfection into 293T cells was performed using PEIMax (40 000 Da; Polysciences Inc., Warrington, PA, USA) [32] or Lipofectamine 3000 (Thermo Fisher Scientific). For transfection using PEI, 1 μg of plasmid DNA was diluted in 100 μL of OPTI‐MEM, followed by the addition of 3 μL of a 1 mg·mL^−1^ PEI solution and vortexing immediately for mixing. The mixture was then spun down using a centrifuge and incubated at 25 °C for 15 min to form the DNA‐PEI complex. After the addition of cultured cells, the medium was replaced after 6 h, and the cells and culture supernatants were collected after the desired incubation time.

**Table 2:** Plasmid list.

Plasmid name	Source	Backbone	Epitope Tag	Selection
pQC.XIP	Clontech			Amp
pQC.HiBiT‐FLAG‐LC3A	–	pQCxIP	N.HiBiT‐FLAG	Amp
pQC.HiBiT‐FLAG‐LC3B	–	pQCxIP	N.HiBiT‐FLAG	Amp
pQC.HiBiT‐FLAG‐LC3C	–	pQCxIP	N.HiBiT‐FLAG	Amp
pQC.HiBiT‐FLAG‐GABARAP	–	pQCxIP	N.HiBiT‐FLAG	Amp
pQC.HiBiT‐FLAG‐GABARAPL1	–	pQCxIP	N.HiBiT‐FLAG	Amp
pQC.HiBiT‐FLAG‐GABARAPL2	–	pQCxIP	N.HiBiT‐FLAG	Amp
pQC.HiBiT‐FLAG‐LC3BΔG	–	pQCxIP	N.HiBiT‐FLAG	Amp
pQC.HiBiT‐FLAG‐LC3BK51A	–	pQCxIP	N.HiBiT‐FLAG	Amp
pQC.HiBiT‐FLAG‐LC3K51AΔG	–	pQCxIP	N.HiBiT‐FLAG	Amp
pQC.HiBiT‐FLAG‐LC3BK51AL53A	–	pQCxIP	N.HiBiT‐FLAG	Amp
pQC.HiBiT‐FLAG‐LC3BK51AL53AΔG	–	pQCxIP	N.HiBiT‐FLAG	Amp
pcDNA3.1.HiBiT‐LC3B	–	pcDNA3.1	N.HiBiT‐FLAG	Amp
pQH.p62‐FLAG‐HiBiT	–	pQCxIP. FLAG‐HiBiT	C.FLAG‐HiBiT	Amp
pQC.HiBiT‐FLAG‐NDP52	–	pQCxIP	N.HiBiT‐FLAG	Amp
pQC.HiBiT‐FLAG‐OPTN	–	pQCxIP	N.HiBiT‐FLAG	Amp
pUA Cas9	Maeda *et al*. [27]			Amp
pUA‐U6p	Maeda *et al*. [27]			Amp
pX459	Addgene #62988			Amp
LAMP1‐mGFP	Addgene #34831		C.mGFP	Kan

### Genome editing using the CRISPR/Cas system

HiBiT‐LC3B KI 293T cells were designed to fuse the HiBiT tag with the N terminus of endogenous LC3B using the CRISPR/Cas system and were established [33]. Next, the CRISPR/Cas system was used to establish HiBiT‐LC3B KI/ATG5 KO 293T cells in which endogenous *ATG5* was knocked out, as well as HiBiT‐LC3B KI/FIP200 KO 293T cells in which endogenous *FIP200* was knocked out. The target sequences of the gRNAs used for genome editing are listed in Table 3. A DNA fragment containing sgRNA under the control of the U6 promoter and Cas9 expression plasmid vector (Cas9T2A‐PurR) was transfected into the cells. To establish knock‐in cells, the repair template DNA with 33‐base homology arms flanking the 33‐base HiBiT tag sequence was cotransfected. The cells were then selected by culturing in a medium containing 1.25 μg·μL^−1^ puromycin (Nacalai Tesque), followed by cloning using the limiting dilution method. The edited genomic region of each clone was amplified by PCR, and genome editing was confirmed by direct sequencing.

**Table 3:** Target sequence for the CRISPR/Cas9 guide RNA.

Name	Targeting exon	sgRNA primer	Target sequence	PCR genotyping primer
LC3B	1	cttgctatttctagctctaaaacACGGCATGGTGCAGGGATCTcggtgtttcgtcctttccacaag	agatccctgcaccatgccgt	PCR Forward primer. cccggagacggcgcggcctg
				PCR Reverse primer. ccggggcccagcacattccc
				PCR Forward primer. aaaaaGGTACCccttcctctgacccctccct
				PCR Reverse primer. aaaaaCTCGAGgcgcagccccgcttcagagc
				sequense primer. aaaaaGGTACCccttcctctgacccctccct
				sequense primer. aaaaaCTCGAGgcgcagccccgcttcagagc
ATG5	2	CACCGTGATATAGCGTGAAACAAGT	acttgtttcacgctatatca	PCR Forward primer. aaaaGGTACCCTAGCAGGTTCATTCATCGTTGC
				PCR Reverse primer. aaaaCTCGAGGCCTCCAAGTTCTTACAGCTTTT
				sequense primer. CGTTGCAAGGATCTGACTAATGC
				sequense primer. aaaaCTCGAGGCCTCCAAGTTCTTACAGCTTTT
FIP200	4	cttgctatttctagctctaaaacggtgttgaatagcaatcttgcggtgtttcgtcctttccacaag	caagattgctattcaacacc	PCR Forward primer. Agggataggaaggaatcttcaca
				PCR Reverse primer. Aaatactgagcgtgcacattg
				sequense primer. Agccagttgttatggaatcctgtt
				sequense primer.aaatactgagcgtgcacattg
Tomm20	5	cttgctatttctagctctaaaaccatcatcttcagccaagctccggtgtttcgtcctttccacaag	catcatcttcagccaagctc	PCR Forward primer. Tctgcctcctttgttaacttgac
				PCR Reverse primer. Gcatatttgcccttattcccc
				sequense primer. Tctgcctcctttgttaacttgac

HiBiT repaire template sequence	
Repaire template	Sequence
HiBiT‐LC3B	cgaaggtgcggcgctgcttgaaggtcttctccgaaatcttcttgaacagccgccagccgctcacGggcatggtgcagggatctgggcggcggcggcggc
HiBiT‐Tomm20	attgtaagtgctcagagcttggctgaagatgatgtgagcggctggcggctgttcaagaagattagcgaatgagaaacaaatgtcaacataataaaatct

### Autophagy flux assay

Autophagy was induced by starvation by replacing the medium with (−) Hank's balanced salt solution (HBSS) (Nacalai Tesque) and culturing for 6 h. Sodium dodecyl sulfate/polyacrylamide gel electrophoresis (SDS/PAGE) and western blotting using LC3 antibodies were performed on the collected cells, and autophagy induction was assessed by detecting higher levels of LC3‐II in starved cells than in cells cultured under nutrient‐rich conditions. Additionally, BafA1 (AdipoGen Life Sciences, Furinsdorf, Switzerland) was added to the medium at a final concentration of 100 nm, and the cells were cultured for 6 h to confirm the inhibition of lysosomal acidification.

### Purification of EVs

The method for the purification of EVs has been previously described [27]. Briefly, depending on the experimental conditions, the culture supernatant of the cells cultured for the desired time was collected and centrifuged at 500 ***g*** for 5 min at 4 °C to remove residual cells. The supernatant was centrifuged at 1200 ***g*** for 5 min at 4 °C to eliminate any remaining cell fragments and debris. The resulting supernatant was centrifuged at 10 000 ***g*** for 30 min, and the obtained pellet was referred to as the 10k precipitate (10k ppt), from which the microvesicle fraction (100–1000 nm in size) was collected. The supernatant from the 10 000 ***g*** centrifugation, referred to as the 10k supernatant (10k sup), was then centrifuged at 100 000 ***g*** for 70 min, after which the pellet was referred to as the 100k precipitate (100k ppt), from which the exosome fraction (50–150 nm in size) was collected. The final supernatant was collected as 100k supernatant (100k sup).

### Measurement of HiBiT‐dependent NanoLuc luciferase activity (HiBiT activity)

A Nano‐Glo HiBiT extracellular detection system (Promega, Madison, WI, USA) was used to measure the HiBiT activity. The LgBiT solution was prepared by mixing the HiBiT extracellular buffer, LgBiT protein, and HiBiT extracellular substrate at a ratio of 100 : 1 : 2. A mixture of 10 μL of the LgBiT solution and 10 μL of the sample to be measured for HiBiT activity was reacted. Luminescence was measured for 1 s using a Varioskan LUX (Thermo Fisher) with a 384‐well white plate (Greiner Bio‐One, Frickenhausen, Germany). For the HiBiT activity measurement using detergent (detergent assay), the Nano‐Glo® HiBiT extracellular detection system (Promega) was used. In this case, 0.1% Triton X‐100 was added to the LgBiT solution to form a detergent + LgBiT solution. For the detergent assay, two LgBiT solutions were prepared: one without detergent (detergent) and one with detergent (detergent +). Measurements were then made. The HiBiT activity in the cell fractions of each cell line was compared using HiBiT activity corrected based on the amount of protein. Protein quantification was performed using Bio‐Rad protein assay dye reagent concentrate (Bio‐Rad, Hercules, CA, USA). HiBiT activity was measured in the culture supernatant and cells, and the secretion rate was calculated by dividing the HiBiT activity in the supernatant by the sum of the HiBiT activities in the cell culture.

### Statistical analysis

Statistical analysis was performed by comparing the means of two groups using Student's *t*‐test. For multiple comparisons, a one‐way analysis of variance (ANOVA) was applied, followed by Dunnett's *post hoc* test for comparison against the control group. In addition, when examining the effects of two independent variables simultaneously, a two‐way analysis of variance (two‐way ANOVA) was conducted. When significant differences were detected, Tukey's *post hoc* test was applied for pairwise multiple comparisons. All analyses were performed using prism 10 (GraphPad Software Inc., Boston, MA, USA). Statistical significance was defined as *P < 0.05, **P < 0.01. ***P < 0.001, and ****P < 0.0001.

## Results

### Establishment of HiBiT knock‐in LC3B 293T cells using the CRISPR/Cas9 system

**Fig. 1:**
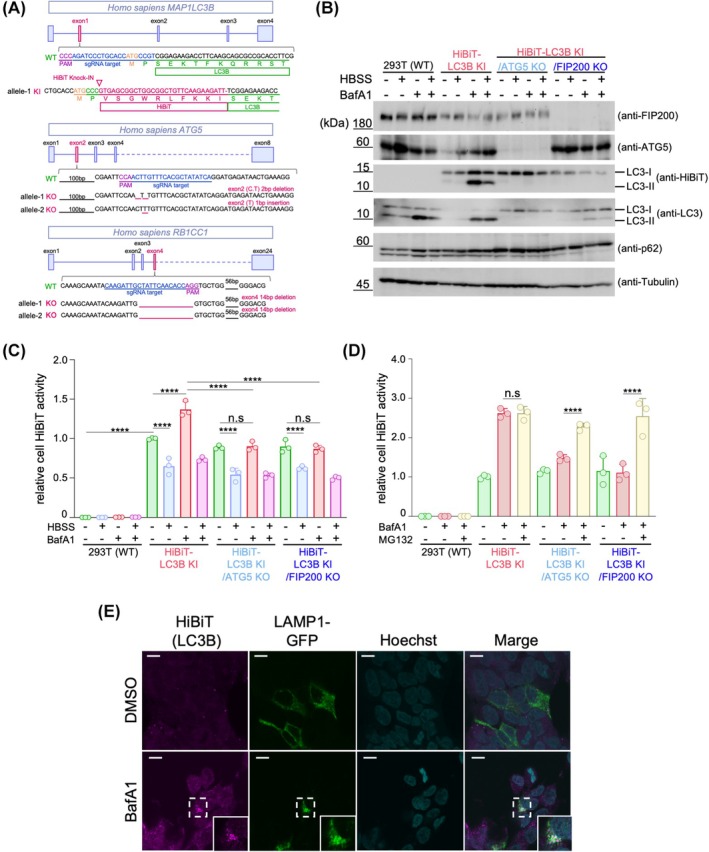
Establishment of HiBiT‐LC3B knock‐in 293T cells. (A) Schematic presentation of the human LC3B genomic locus. The inserted HiBiT tag sequence is shown in red. (B) Detection of HiBiT‐LC3B, ATG5, or FIP200 using western blotting. WT, HiBiT‐LC3B‐KI, HiBiT‐LC3B KI/ATG5 KO, or HiBiT‐LC3B KI/FIP200 KO 293T cells were treated with or without 100 nm of bafilomycin A1 (BafA1), or the medium was replaced with Hank's balanced salt solution (HBSS) for 6 h. The cells were then lysed, and the lysate was used for western blotting to measure the amount of endogenous FIP200 (1st panel), ATG5 (2nd panel), HiBiT fused LC3B (3rd panel), LC3B (4th panel), p62 (5th panel), and alpha‐tubulin (6th panel) using antibodies recognizing each endogenous protein or anti‐HiBiT antibodies. (C) HiBiT‐dependent NanoLuc luciferase activities (HiBiT activity) of the cell lysate from WT or KI cells. HiBiT activities in the cell lysate used in (B) were measured. Data are presented as mean ± SD of three technical replicates (*n* = 3). Experiments were independently repeated at least twice with similar results. ****P < 0.0001; n.s., not significant (two‐way ANOVA and Tukey's multiple comparisons test). (D) Effect of MG132, a proteasome inhibitor, on the accumulation of LC3B. HiBiT‐LC3B KI, HiBiT‐LC3B KI ATG5 KO, or HiBiT‐LC3B KI FIP200 KO 293T cells were treated with 100 nm BafA1 and/or 10 μm MG132, or mock for 12 h. The cells were then lysed, and HiBiT activities were measured. Data are presented as mean ± SD of three technical replicates (*n* = 3). Experiments were independently repeated at least twice with similar results. ****P < 0.0001; n.s., not significant (two‐way ANOVA and Tukey's multiple comparisons test). (E) Bafilomycin A1 induces lysosomal accumulation of HiBiT‐LC3B. HiBiT‐LC3B KI 293T cells were transiently transfected with LAMP1‐GFP (green; lysosomal marker) and treated for 6 h with 100 nm BafA1 (bottom panels) or without (top panels). Cells were fixed and immunostained with an anti‐HiBiT antibody (magenta; detecting HiBiT‐LC3B) and Hoechst 33342 (cyan; nuclei). Insets show magnified views of the boxed regions. Scale bar = 10 μm.

In this study, we introduced the HiBiT tag system to analyze the behavior of the autophagosome marker LC3 both intracellularly and extracellularly. We established a HiBiT knock‐in LC3B 293T cell line (HiBiT‐LC3B KI cells) by designing a HiBiT tag fused to the N terminus of endogenous LC3B via genome editing using CRISPR/Cas9 [29, 33] (Fig. 1A). Furthermore, we performed genome editing to knock out the gene encoding ATG5, a factor involved in LC3 lipid modification, in HiBiT knock‐in LC3B 293T cells. We established an *ATG5* knockout HiBiT knock‐in LC3B 293T cell line (HiBiT‐LC3B KI/ATG5 KO). Recent studies have shown that LC3 lipid modification is not essential for autophagosome formation. Therefore, we also established an *FIP200* knockout HiBiT knock‐in LC3B 293T cell line (HiBiT‐LC3B KI/FIP200 KO cells) in which *FIP200*, an upstream factor involved in autophagosome formation, was knocked out. Western blot analysis under starvation conditions or after treatment with bafilomycin A1 (BafA1), an inhibitor of V‐ATPase that prevents lysosomal acidification [34], showed that the HiBiT tag was detected at the same molecular weight as LC3B only in HiBiT knock‐in cell lines (Fig. 1B). This confirmed that the HiBiT tag was expressed as a fusion protein with LC3B in HiBiT knock‐in cells. No other bands were detected, confirming the absence of off‐target HiBiT fusion proteins. Additionally, in *ATG5* knockout cells, LC3‐II formation was not observed during starvation or after BafA1 treatment. In *FIP200* knockout cells, significant inhibition of LC3‐II formation was observed under these conditions. Accumulation of the autophagy substrate p62 [35, 36] was also observed in *ATG5* or *FIP200* knockout cells compared to that in wild‐type (WT) cells. These results indicate that LC3 lipid modification was inhibited by knocking out *ATG5* or *FIP200*, leading to defects in the autophagy pathway.

Next, we quantified the HiBiT‐dependent NanoLuc luciferase activity (HiBiT activity) in these cells (Fig. 1C). The knock‐in cells showed significantly higher HiBiT activity than WT cells. When HiBiT activity was examined after 6 h of starvation, a decrease in intracellular HiBiT activity was observed compared with that in untreated HiBiT‐LC3B KI cells. This was confirmed in *ATG5* knockout and *FIP200* knockout cells. In contrast, treatment with BafA1 increased HiBiT activity compared to untreated HiBiT‐LC3B KI cells, suggesting that LC3B degradation in the lysosomes was inhibited. This increase in HiBiT activity was not observed in *ATG5* or *FIP200* knockout cells, suggesting that the lysosomal delivery system is deficient in these cells. These results indicate that intracellular levels of LC3 change in an autophagy‐ or lysosomal activity‐dependent manner.

Jia and Bonifacino [37] previously reported that LC3 can be degraded by the proteasome through ubiquitination. To examine the role of the proteasome in LC3 degradation under autophagy‐deficient conditions, we treated cells with the proteasome inhibitor MG132 alongside BafA1 and measured LC3 accumulation. As shown in Fig. 1D, MG132 treatment led to a marked increase in HiBiT activity in both ATG5 KO and FIP200 KO HiBiT‐LC3B knock‐in cells. These results indicate that, in the absence of autophagy, LC3 is at least partly degraded via the proteasomal pathway.

In immunofluorescence analysis, HiBiT‐LC3B exhibited a diffuse cytoplasmic distribution under basal conditions. However, following treatment with bafilomycin A1 (BafA1), the HiBiT‐LC3B signal accumulated in distinct compartments that colocalized with the lysosomal marker LAMP1‐GFP (Fig. 1E). This accumulation upon lysosomal inhibition indicates that HiBiT‐LC3B is targeted to lysosomes, consistent with the behavior of untagged LC3. These results confirm that the HiBiT tag does not alter the native localization or trafficking of LC3.

### Fractionation of HiBiT‐LC3B secreted into the culture supernatant and verification of the presence or absence of encapsulation by lipid membranes

**Fig. 2:**
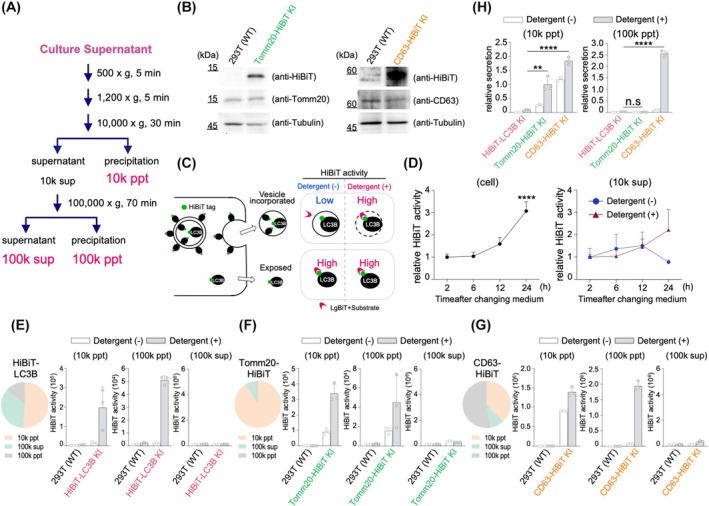
Detection of HiBiT‐LC3B in the culture supernatant. (A) Diagram showing the protocol for the fractionation of cell culture supernatants via serial centrifugation. (B) Detection of TOMM20‐HiBiT or CD63‐HiBiT using western blotting. WT, TOMM20‐HiBiT KI, and CD63‐HiBiT KI 293T cells were lysed and used for western blotting to measure the amount of endogenous TOMM20 (left middle panel), CD63 (right middle panel), alpha‐tubulin (bottom panels), HiBiT fused with TOMM20 (left upper panel), or HiBiT fused with CD63 (right upper panel) using antibodies recognizing each endogenous protein or anti‐HiBiT antibodies. (C) Schematic showing the detergent sensitivity assay. By measuring HiBiT activities in both the presence and absence of detergent, we determined whether a phospholipid membrane surrounds the HiBiT tag. (D) Increase in the HiBiT‐LC3B content in the cell and culture supernatants. HiBiT activities of cell lysate (left graph) or culture supernatant (right graph) at the indicated time were plotted. In the culture supernatant, HiBiT activity was measured with or without 0.1% Triton X‐100. Data are presented as mean ± SD of three technical replicates (*n* = 3). Experiments were independently repeated at least twice with similar results. ****P < 0.0001 (One‐way ANOVA and Dunnett's multiple comparisons test). (E) Distribution of secreted HiBiT‐LC3B in each centrifugation fraction. The HiBiT activities of the 10 000 ***g*** precipitate (10k ppt, 2nd graph), 100 000 ***g*** precipitate (100k ppt, 3rd graph), and 100 000 ***g*** supernatant (100k sup, 4th graph) fractions were measured at 48 h. LCB‐KI cell culture supernatants were measured with or without 0.1% Triton X‐100 (detergent). The distribution of HiBiT activities in each fraction is shown in the left pie chart. Data are presented as mean ± SD of three technical replicates (*n* = 3). Experiments were independently repeated at least twice with similar results. (F) Distribution of secreted HiBiT‐TOMM20 in each centrifugation fraction. Data are presented as mean ± SD of three technical replicates (*n* = 3). Experiments were independently repeated at least twice with similar results. (G) Distribution of secreted HiBiT‐CD63 in each centrifugation fraction. Data are presented as mean ± SD of three technical replicates (*n* = 3). Experiments were independently repeated at least twice with similar results. (H) Comparison of the secretion ratio between each HiBiT‐KI cell. The secretion ratios of TOMM20‐HiBiT, CD63‐HiBiT, and HiBiT‐LC3B in the 10k ppt fraction (left graph) or 100k ppt fraction (right graph) are shown. These data are derived from the experiment shown in (E–G). Data are presented as mean ± SD of three technical replicates (*n* = 3). Experiments were independently repeated at least twice with similar results. **P < 0.01 and ****P < 0.0001; n.s., not significant (one‐way ANOVA and Dunnett's multiple comparisons test).

Using established HiBiT knock‐in cells, we detected the release of HiBiT‐LC3B into the culture supernatant. The supernatant was fractionated to analyze the HiBiT‐LC3B in the culture supernatant. Following the purification method for EVs, such as exosomes [27], we fractionated the culture supernatant via low‐speed centrifugation, obtaining a 10 000 ***g*** precipitate fraction (10k ppt) and a 10 000 ***g*** supernatant fraction (10k sup). The 10k sup fraction was further fractionated via ultracentrifugation into a 100 000 ***g*** precipitate fraction (100k ppt) and a 100 000 ***g*** supernatant fraction (100k sup), and the HiBiT activity in each fraction was measured (Fig. 2A).

TOMM20, a mitochondrial outer membrane protein [38, 39], was used as the control. A TOMM20‐HiBiT knock‐in 293T cell line, in which a HiBiT tag was fused to the C terminus of endogenous TOMM20 via genome editing, was established and used in the experiments. Additionally, a CD63‐HiBiT knock‐in 293T cell line in which HiBiT was knocked in at the C terminus of CD63 (a factor incorporated into EVs) [27] was used for comparison (Fig. 2B).

In this study, HiBiT activity was detected using a detergent to determine the presence of HiBiT‐LC3B within lipid membrane vesicles. By measuring activity in the presence and absence of 0.1% Triton‐X 100, we determined whether the HiBiT tag was encapsulated within the lipid membranes, thereby confirming whether HiBiT‐LC3B was secreted in the form of membrane vesicles (Fig. 2C). HiBiT activity in both the cells and the 10k sup fraction of the culture supernatant gradually increased after the medium was changed to standard medium (Fig. 2D). In the culture supernatant, HiBiT activity increased only in the presence of the detergent, but not in its absence, suggesting that vesicle‐incorporated HiBiT‐LC3B, rather than uncoated naked HiBiT‐LC3B, was actively secreted. These results indicate that even at a steady state, LC3B molecules are secreted in membrane vesicles.

Each knock‐in cell line was cultured in a standard medium for 48 h; the culture supernatants were collected and centrifuged according to the method shown in Fig. 2A to examine the distribution of HiBiT activity in the collected fractions. HiBiT‐LC3B secreted extracellularly was most abundantly detected in the 10k ppt fraction (Fig. 2E). Furthermore, when the detergent dependency of HiBiT activity in each fraction was investigated, HiBiT‐LC3B was detected at higher levels in the presence of detergent in the 10k ppt fraction, suggesting that HiBiT‐LC3B mainly exists in membrane vesicles in this fraction. HiBiT activity was also detected, albeit at low levels, in the 100k ppt fraction, showing detergent dependence similar to that of the 10k ppt fraction. In contrast, HiBiT‐LC3B in the 100k sup fraction was detected, regardless of the presence or absence of detergent, suggesting that HiBiT‐LC3B in this fraction may exist in an exposed form without being encapsulated in the lipid membrane.

Similar analyses were performed using the TOMM20‐HiBiT KI and CD63‐HiBiT KI cells. TOMM20, secreted extracellularly, was most abundant in the 10k ppt fraction, identical to HiBiT‐LC3B. Additionally, most TOMM20 in this fraction was detected in a detergent‐dependent manner, indicating that TOMM20, similar to LC3B, was encapsulated in lipid membranes. In contrast, TOMM20 was detected in the 100k sup fraction, albeit in small amounts, regardless of the presence of detergent, indicating that TOMM20 in this fraction was not membrane‐bound (Fig. 2F). CD63 was most abundantly detected in the 100k ppt fraction, with a certain amount found in the 10k ppt and 100k sup fractions (Fig. 2G). Furthermore, as CD63 was detected at higher levels in the presence of detergents in all fractions, we suggest that most of the secreted CD63 was released extracellularly in the form of membrane vesicles.

HiBiT activity in the culture supernatant of each knock‐in cell line was compared in both the 10k ppt and 100k ppt fractions (Fig. 2H). In the 10k ppt fraction, the extracellular secretion rate of LC3B was approximately 20‐fold lower than that of CD63. In the 100k ppt fraction, the secretion rates of both LC3B and TOMM20 were similarly low and significantly lower than that of CD63 (Fig. 2H). These results indicate that the extracellular secretion of LC3B and TOMM20 under steady‐state conditions was minimal compared to the amount of CD63 present in the EVs.

### Detection of HiBiT‐LC3B secretion from cells with disrupted lysosomal function

**Fig. 3:**
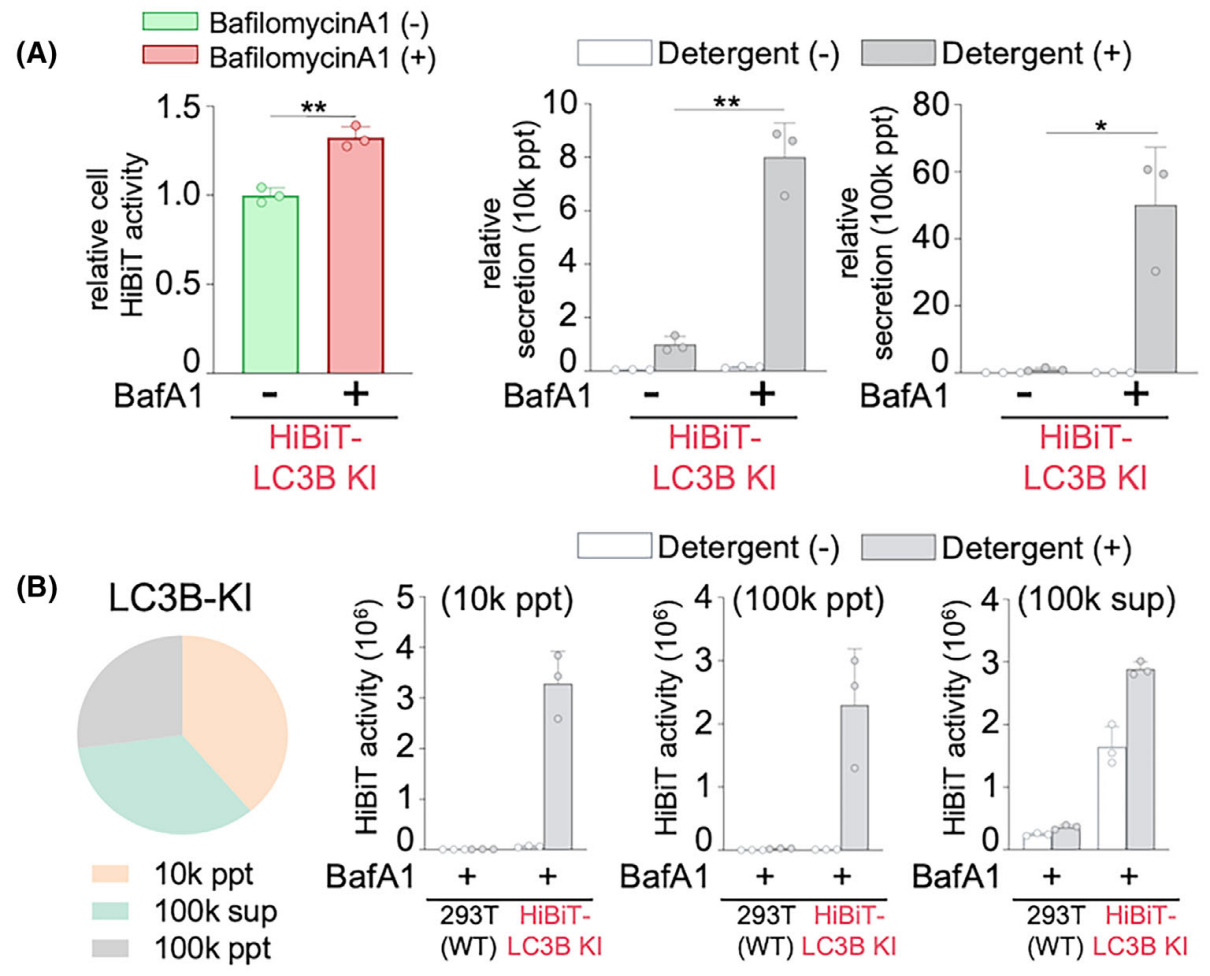
Bafilomycin A1 treatment significantly enhances HiBiT‐LC3B secretion. (A) Effect of bafilomycin A1 treatment on HiBiT‐LC3B secretion. WT or HiBiT‐LC3B‐KI 293T cells were treated with or without 100 nm of BafA1 for 6 h. Then, the cells were lysed and HiBiT activity was measured (left graph). The 10k ppt (middle graph) or 100k ppt (right graph) fractions of the culture supernatants from the HiBiT‐LC3B KI cells were collected, and HiBiT activities were measured in the presence or absence of detergent; the secretion rate was calculated by dividing the HiBiT activity of the 10k ppt fraction by the sum of the HiBiT activity of the cell culture. Data are presented as mean ± SD of three technical replicates (*n* = 3). Experiments were independently repeated at least twice with similar results. *P < 0.05 and **P < 0.01 (Student's t‐test). (B) Distribution of secreted HiBiT‐LC3B from BafA1‐treated cells. After 6 h, BafA1 (100 nm)‐treated HiBiT‐LC3B KI cells were harvested and fractionated via serial centrifugation as shown in Fig. 2A. HiBiT activities of the 10k ppt (2nd graph), 100k ppt (3rd graph), and 100k supernatant (4th graph) fractions were measured with or without 0.1% Triton X‐100. The distribution of HiBiT activities in each fraction is shown in the pie chart on the left. Data are presented as mean ± SD of three technical replicates (*n* = 3). Experiments were independently repeated at least twice with similar results.

Next, we investigated the effects of BafA1 treatment on HiBiT secretion in HiBiT‐LC3B cells. After treating cells with BafA1, HiBiT activity was measured in each fraction after a 6‐h incubation period. BafA1 treatment significantly elevated the secretion rate of LC3B in both the 10k ppt and 100k ppt fractions (Fig. 3A). These results indicate that the disruption of lysosomal function facilitates the secretion of LC3B, suggesting that LC3B, which is typically transported to and degraded in lysosomes, is redirected for extracellular release.

To further elucidate the conditions under which LC3B was secreted extracellularly during BafA1 treatment, we assessed HiBiT activity in the supernatants and pellets of each centrifuged fraction. As shown in Fig. 3B, the amount of LC3B after BafA1 treatment was also increased in the 100k ppt fraction. Furthermore, the detergent‐dependent detection of LC3B in this fraction indicated that LC3B was encapsulated and subsequently secreted. These results suggest that LC3B is released extracellularly within comparatively small vesicles owing to lysosomal dysfunction.

### Secretion of the LC3 family proteins

**Fig. 4:**
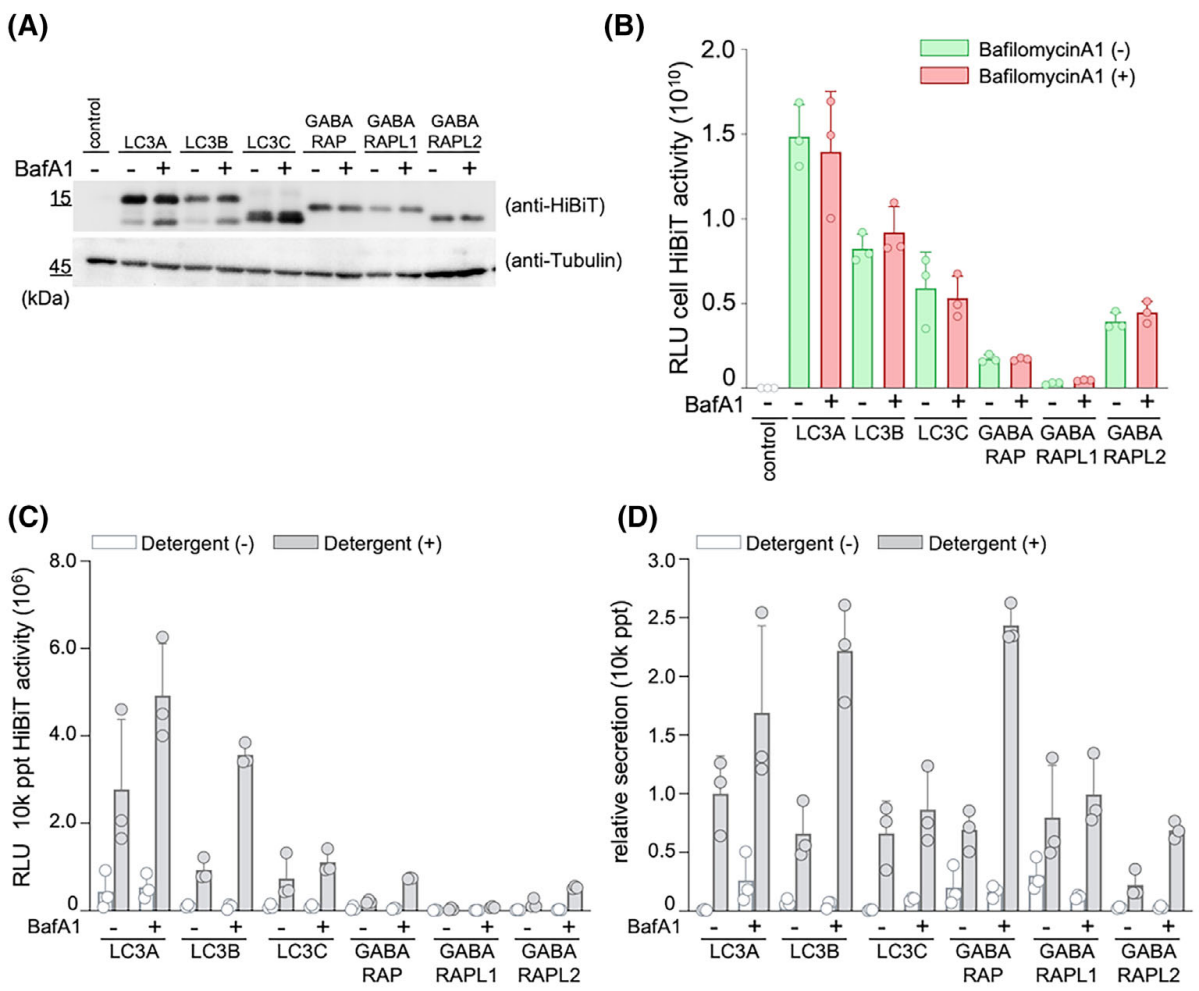
Secretion of the LC3 family of proteins. (A) Expression of HiBiT‐tagged LC3 family proteins. Empty or LC3 family protein‐expressing vectors were transfected into the 293T cells. After 24 h of transfection, the expression levels of LC3 family proteins (top panel) and alpha‐tubulin (bottom panel) were measured using western blotting with anti‐HiBiT or anti‐alpha‐tubulin antibodies, respectively. (B) Detection of HiBiT‐tagged LC3 family proteins. The cells were treated with or without 100 nm of BafA1 for 6 h after plasmid transfection. Cells were harvested, and HiBiT activities were measured. Data are presented as mean ± SD of three technical replicates (*n* = 3). Experiments were independently repeated at least twice with similar results. (C) Detection of the secreted HiBiT‐tagged LC3 family proteins. Culture supernatants in the experiment of (B) were harvested, and HiBiT activities were measured in the presence or absence of 0.1% Triton X‐100. Data are presented as mean ± SD of three technical replicates (*n* = 3). Experiments were independently repeated at least twice with similar results. (D) The secretion rate of LC3 family proteins. The secretion rate was calculated by dividing the HiBiT activity of the 10k supernatant fraction by the sum of the HiBiT activity of the cell culture. Data are presented as mean ± SD of three technical replicates (*n* = 3). Experiments were independently repeated at least twice with similar results.

LC3, a homolog of yeast ATG8, is comprised of six family members [11, 12, 40]. We analyzed whether the results obtained from the established HiBiT knock‐in cells for LC3B could be applied to other members of the LC3 family. To this end, we constructed plasmid vectors in which the HiBiT tag was fused to each LC3 family member, and performed transient expression analysis (Fig. 4A). A total of 293T cells were transfected with each LC3 expression vector, and the secretion rates in the presence and absence of BafA1 treatment, as well as the detergent dependency of HiBiT activity in the supernatant, were examined (Fig. 4B,C). The results showed that the secretion rates for the 6‐h cultivation periods were similar to each other, and these values were comparable to the secretion rate of LC3B in HiBiT‐LC3B KI cells. In all cases, the secretion rate increased with BafA1 treatment and in a detergent‐dependent manner. These results suggested that all LC3 family proteins are secreted extracellularly, similar to LC3B.

### Role of LC3B lipidation in LC3B secretion

During autophagy induction, LC3 is conjugated with PE by a conjugation system including ATG5 and anchored to the autophagosome membrane [9, 41]. In this study, we examined the involvement of LC3B lipidation in LC3B secretion from *ATG5* knockout cells.

**Fig. 5:**
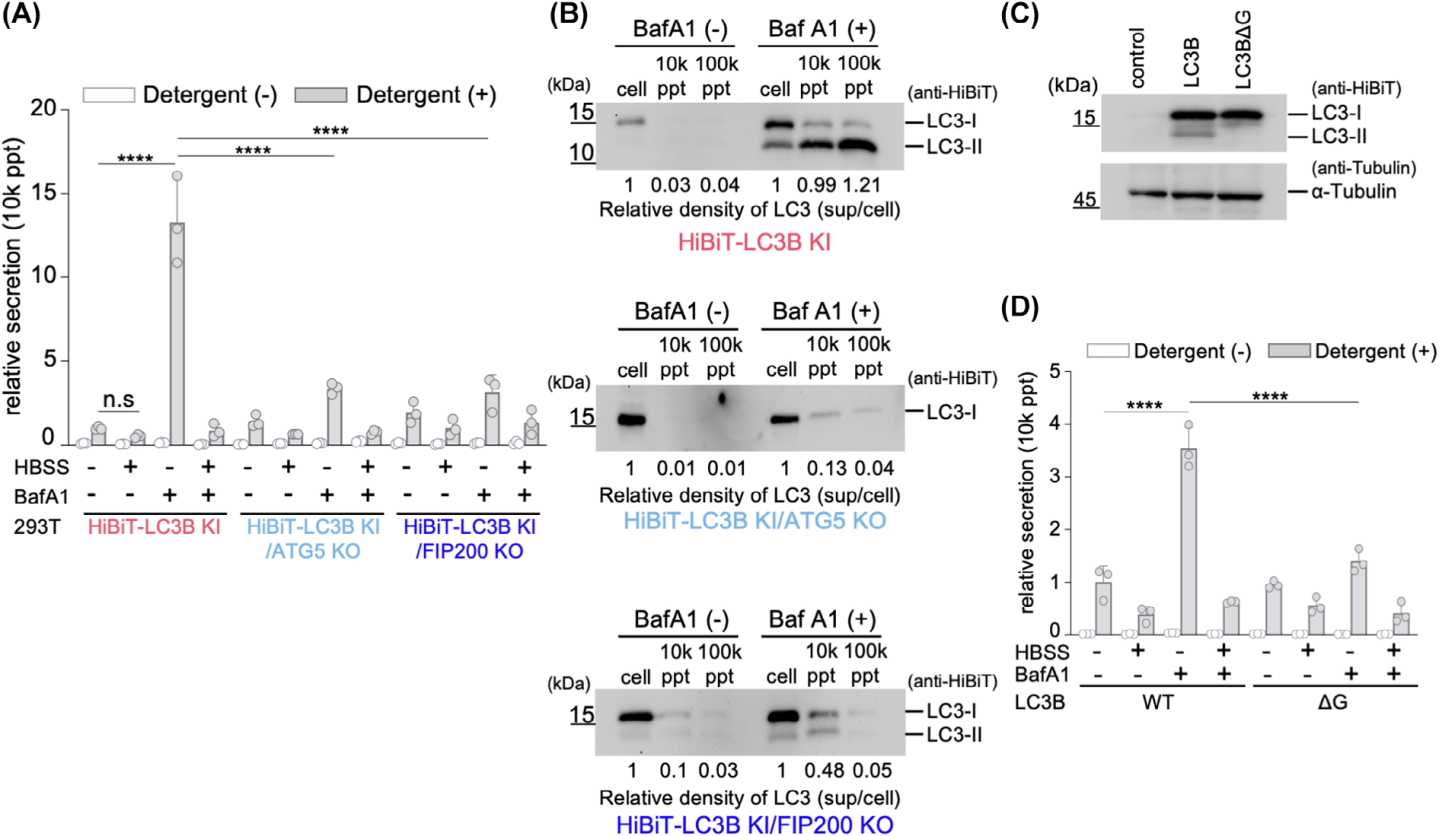
Lipid modification of LC3 and autophagosome formation are partially involved in LC3 secretion. (A) Secretion of HiBiT‐LC3B from HiBiT‐LC3B KI, HiBiT‐LC3B KI/ATG5 KO, or HiBiT‐LC3B KI/FIP200 KO 293T cells. The HiBiT‐LC3B KI cells were treated with or without 100 nm of BafA1, or the medium was replaced with HBSS for 6 h. Cells and culture supernatants were harvested, and HiBiT activities were measured in the presence or absence of 0.1% Triton X‐100. The secretion rate was calculated by dividing the HiBiT activity of the 10k ppt fraction by the sum of the HiBiT activity of the cell culture. Data are presented as mean ± SD of three technical replicates (*n* = 3). Experiments were independently repeated at least twice with similar results. ****P < 0.0001; n.s., not significant (two‐way ANOVA and Tukey's multiple comparisons test). (B) Detection of secreted HiBiT‐LC3B using western blotting. After 12 h of cultivation with or without 100 nm of BafA1, the cells and culture supernatants of HiBIT‐LC3B KI (top panel), HiBiT‐LC3B KI/ATG5 KO (middle panel), and HiBiT‐LC3B KI/FIP200 KO (bottom panel) were harvested. Then, the culture supernatants were centrifuged using the protocol shown in Fig. 2A. HiBiT‐LC3B in each fraction was detected via western blotting using an anti‐HiBiT tag antibody. The numbers below the panels represent the relative amounts of LC3 proteins in the 10k or 100k ppt fractions compared to those in the cell fraction, calculated based on band intensity. (C) Expression of HiBiT‐tagged LC3B deltaG mutant. Empty vector, HiBiT‐LC3B WT, and HiBiT‐LC3B deltaG mutant expressing vectors were transiently expressed in 293T cells, and the expression level of HiBiT‐LC3B was measured using western blotting with an anti‐HiBiT antibody. (D) Secretion from the HiBiT‐tagged LC3B deltaG mutant. HiBiT‐LC3B WT or HiBiT‐LC3B deltaG mutant were transiently expressed in 293T cells. After 24 h of cultivation, the cells were treated with or without 100 nm BafA1, or the medium was replaced with HBSS and incubated for 6 h. Cells and culture supernatants were harvested, and HiBiT activities were measured in the presence or absence of 0.1% Triton X‐100. The secretion rate was calculated by dividing the HiBiT activity of the 10k ppt fraction by the sum of the HiBiT activity of the cell culture. Data are presented as mean ± SD of three technical replicates (*n* = 3). Experiments were independently repeated at least twice with similar results. ****P < 0.0001 (two‐way ANOVA and Tukey's multiple comparisons test).

Using HiBiT knock‐in cells, we calculated the rate of extracellular LC3B secretion during starvation and after BafA1 treatment. The secretion rate of HiBiT‐LC3B in the culture supernatant of cells treated with BafA1 was approximately 13‐fold higher than that in the supernatant of untreated cells. In contrast, the amount of HiBiT‐LC3B secreted extracellularly under nutrient starvation conditions was lower than that under steady‐state conditions. In addition, the increase in secretion because of BafA1 addition decreased significantly under starvation conditions. These results suggested that the autophagic secretion pathway changes depending on the environment surrounding the cells (Fig. 5A). Furthermore, compared to the WT, changes in the secretion rate of LC3B were not observed under steady‐state conditions with the *ATG5* knockout; however, BafA1‐dependent extracellular secretion was significantly reduced. These results indicated that BafA1‐dependent LC3B secretion depends on LC3B lipidation or the autophagy pathway.

Next, we assessed whether extracellularly secreted LC3B was LC3B‐I or LC3B‐II. Using knock‐in cells, we detected endogenous LC3B secretion using western blotting (Fig. 5B). HiBiT‐LC3B KI cells were cultured for 48 h, followed by a 12‐h treatment with or without BafA1, and then collected at 10k ppt and 100k ppt. In the analysis of HiBiT‐LC3B KI/ATG5 KO cells, HiBiT‐LC3B was detected in the culture supernatant, although its concentration was lower than that in WT cells. This suggests that LC3B secretion depends on autophagosome formation or the lipid modification of LC3B.

In HiBiT‐LC3B KI cells treated with BafA1, both LC3B‐I and LC3B‐II were detected in culture supernatants. However, because the amount of LC3B‐II detected was higher than that of LC3B‐I, the secreted LC3B was primarily of the type II form (Fig. 5B). In contrast, in the culture supernatant of HiBiT‐LC3B KI/ATG5 KO cells treated with BafA1, LC3B‐I was still detected. These results suggest that in addition to LC3B‐II, which is anchored to the isolation membrane, LC3B‐I, which is not anchored to the isolation membrane, is secreted. Thus, the extracellular LC3B consists of a mixture of lipidated LC3B‐II and nonlipidated LC3B‐I.

To further investigate lipidation‐dependent secretion of LC3B, we analyzed LC3B mutants. We examined the secretion of a mutant (ΔG) lacking the amino acids from the glycine residue at the C‐terminal lipidation site [42] (Fig. 5C). When LC3BΔG was expressed in 293T cells and the change in secretion rate was examined after BafA1 treatment, BafA1‐dependent secretion of HiBiT‐LC3B was found to be suppressed (Fig. 5D). This confirmed that C‐terminal lipidation is essential for BafA1‐dependent secretion of LC3B.

Notably, in both experiments using *ATG5* knockout cells and the ΔG mutant, a certain amount of HiBiT‐LC3B secretion was observed. Additionally, the HiBiT activity detected in the culture supernatant was sensitive to detergent treatment (Fig. 5A,D). These results suggest that LC3B‐I, similar to LC3B‐II, is secreted extracellularly and is enclosed in a phospholipid membrane.

### Role of autophagosome formation in LC3B secretion

There are reports that ATG5‐dependent lipidation of LC3B is not essential for autophagosome formation [43, 44]. Therefore, we investigated the role of autophagosome formation in LC3B secretion using *FIP200* knockout cells, which are required for autophagosome formation (Fig. 5A). The results showed that in the *FIP200* knockout cells, as with the *ATG5* knockout cells, BafA1‐dependent secretion was reduced compared with that in WT cells (Fig. 5A,B). These findings indicate that autophagosome formation is necessary for BafA1‐dependent secretion of LC3B. However, the secretion rate of HiBiT‐LC3B under steady‐state conditions did not differ significantly between *FIP200* knockout and WT cells. These results indicate the existence of multiple secretion pathways for LC3B, with some LC3B being secreted into vesicles without undergoing lipidation, independent of the autophagy pathway.

### Role of autophagy receptor binding in LC3B secretion

**Fig. 6:**
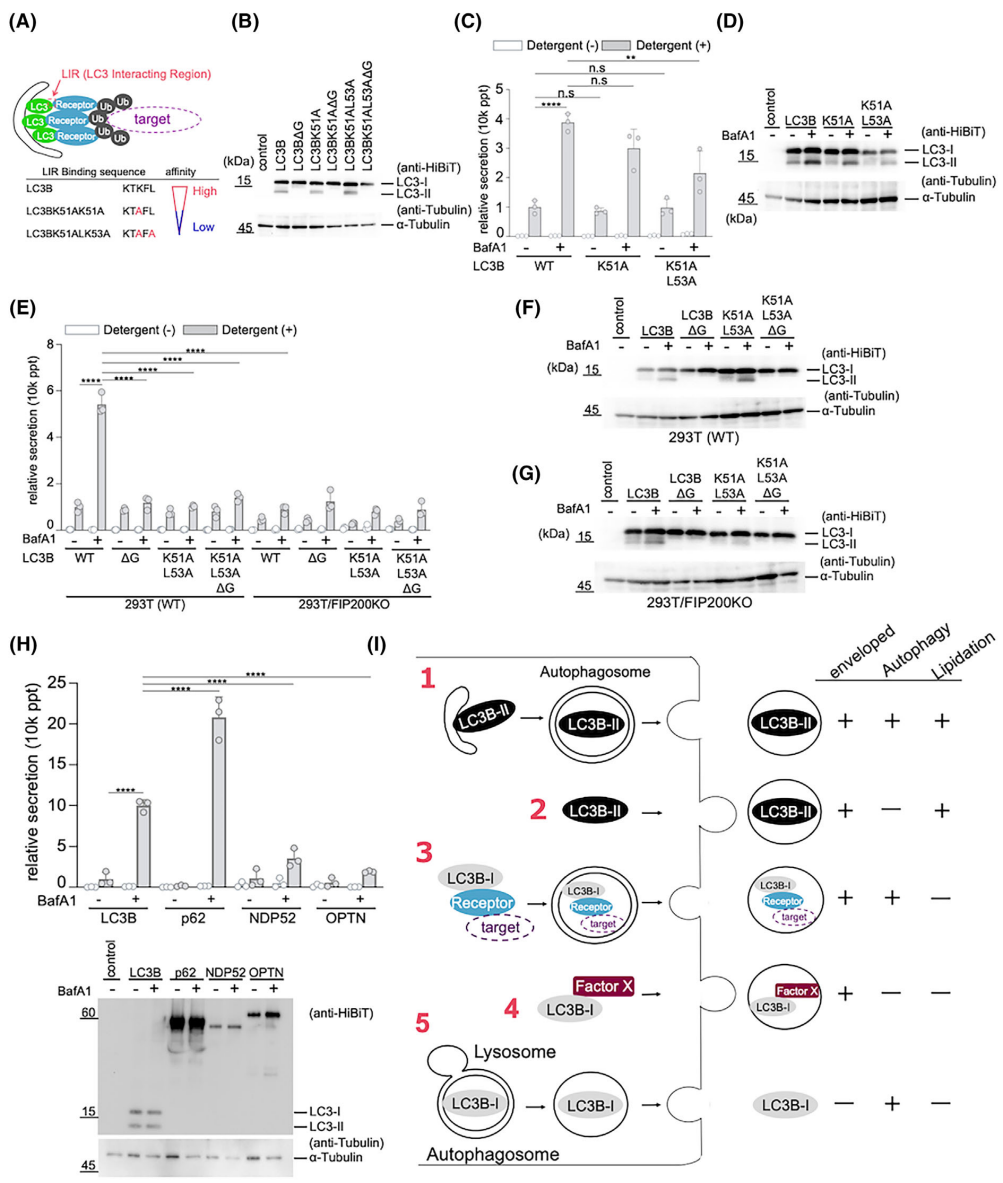
Receptor binding of LC3 is also involved in LC3 secretion. (A) Schematic presentation of LC3‐receptor interaction‐mediated selective autophagy. (B) Expression levels of HiBiT‐tagged LC3B WT or LC3B mutants. Indicated vectors were transfected into the 293T cells. After 24 h of cultivation, HiBiT‐LC3B proteins (top panel) or alpha‐tubulin (bottom panel) in cells were detected using western blotting. (C) Secretion of receptor‐binding‐deficient LC3B mutants. HiBiT‐LC3B WT or mutants were transiently expressed in 293T cells. After 24 h of cultivation, the cells were treated for 6 h with or without 100 nm of BafA1. Cells and culture supernatants were harvested, and HiBiT activities were measured in the presence or absence of 0.1% Triton X‐100. The secretion rate was calculated. Data are presented as mean ± SD of three technical replicates (*n* = 3). Experiments were independently repeated at least twice with similar results. **P < 0.01, ****P < 0.0001; n.s., not significant (two‐way ANOVA and Tukey's multiple comparisons test). (D) LC3‐II formation of receptor‐binding‐deficient LC3B mutants. After 24 h of transfection, the cells were treated with or without 100 nm BafA1 for 6 h, followed by harvesting. HiBiT‐LC3B proteins (top panel) or alpha‐tubulin (bottom panel) were then detected using western blotting with the same protocol shown in (B). (E) Secretion of receptor‐binding‐deficient and/or delta G LC3B mutants. Each vector was transfected into WT or FIP200 KO cells. After 24 h of transfection, the cells were treated with or without 100 nm BafA1 for 6 h, then harvested, and their HiBiT activities were detected. The secretion rate of HiBiT‐LC3B was calculated using the same protocol as in (C). Data are presented as mean ± SD of three technical replicates (*n* = 3). Experiments were independently repeated at least twice with similar results. ****P < 0.0001 (Two‐way ANOVA and Tukey's multiple comparisons test). (F) LC3‐II formation of receptor‐binding‐deficient and or delta G LC3B mutants in WT 293T cells. Western blotting was performed using the same protocol as shown in (B). (G) LC3‐II formation of receptor‐binding‐deficient and or delta G LC3B mutants in *FIP200* KO 293T cells. (H) Secretion of autophagy receptors. Each receptor was transiently expressed in 293T cells. After 24 h of cultivation, the cells were treated with or without 100 nm of BafA1 and incubated for 6 h. Cells and culture supernatants were harvested, and HiBiT activities were measured in the presence or absence of 0.1% Triton X‐100. The secretion rate was calculated. Data are presented as mean ± SD of three technical replicates Data are presented as mean ± SD of three technical replicates (*n* = 3). Experiments were independently repeated at least twice with similar results. ****P < 0.0001 (Two‐way ANOVA and Tukey's multiple comparisons test). The expression level of HiBiT‐tagged autophagy receptors and alpha‐tubulin in cells was confirmed by western blotting. (I) Schematic presentation showing the possible LC3B secretion pathways. LC3B could be secreted via the following five different pathways: (1) autophagy secretion, (2) autophagy‐independent but lipid modification‐dependent secretion, (3) receptor binding‐dependent secretion, (4) autophagy, lipid modification‐independent secretion, and (5) lysosome secretion.

We further analyzed the secretion pathway of LC3B‐I. During selective autophagy, LC3B binds to receptor molecules via the LC3‐interacting region (LIR; W/F/YxxL/I/V consensus) [45, 46]. Replacing Lys 51 in LC3B with alanine reduced its affinity for receptor molecules [47]. Additionally, a mutant in which both Lys 51 and Leu 53 were substituted with alanine showed an even lower affinity for LIR (Fig. 6A,B) [47]. Using this low‐LIR affinity mutant, we examined the role of receptor binding in LC3 secretion.

LC3B‐K51A and LC3B‐K51A/L53A mutants were expressed in 293T cells, and the extracellular secretion rate and detergent sensitivity of HiBiT‐LC3B were determined in the presence or absence of BafA1. As shown in Fig. 6C, compared with WT LC3B, the secretion of LC3B‐K51A/L53A was reduced under both BafA1‐treated conditions. This trend was also observed in the absence of BafA1, although the difference was not significant. These results indicated that binding to receptor molecules is involved in LC3B secretion.

Since these receptor‐binding‐deficient mutations resulted in the accumulation of type II LC3 upon BafA1 treatment (Fig. 6D), we created additional mutants, LC3B‐K51A/ΔG and LC3B‐K51A/L53A/ΔG, with lipidation‐deficient mutations to further investigate the role of receptor binding. The results showed that the secretion rate of WT cells was lower than that of either the receptor‐binding‐deficient or lipidation‐deficient single mutants (Fig. 6E,F). These results suggest that part of the secretion of nonlipidated LC3B occurs via receptor binding.

To investigate whether the receptor‐dependent secretion of LC3B‐I is dependent on the autophagy pathway, we expressed LC3B‐K51A/ΔG and LC3B‐K51A/L53A/ΔG in FIP200 KO cells and examined the secretion rates of these mutants. The results showed that the secretion rates of both LIR low‐affinity mutants and their lipidation‐deficient mutants were lower than those of WT cells. In FIP200 KO cells, the secretion rates of these mutants were further decreased compared to those in WT cells (Fig. 6E,G). These results suggested that these mutants were partially secreted via autophagy.

To investigate whether autophagy receptors are secreted, we expressed HiBiT‐tagged versions of p62 (Bjørkøy *et al*., [48]), NDP52 (Thurston *et al*., [49]), and optineurin (Wild *et al*., [50]) in cells and measured HiBiT activity in both cell lysates and culture supernatants. As shown in Fig. 6H, HiBiT activity was primarily detected in the supernatant after detergent treatment, indicating that these receptors are secreted in a membrane‐enclosed form, similar to LC3. This finding suggests that they are likely released through the secretory autophagy pathway.

## Discussion

In this study, we established an experimental system for the high‐sensitivity detection of LC3B by fusing a HiBiT tag to LC3B. This system enables easy detection of minute amounts of extracellularly secreted LC3B, which was previously challenging to detect and quantify. Consequently, we elucidated the changes in LC3B secretion over short periods under nutrient starvation and lysosomal dysfunction conditions. Furthermore, this system enabled the high‐sensitivity measurement of autophagic activity within the cells (Fig. 1). Generally, methods for measuring autophagic activity include assessing the lipidation of LC3B [9, 41], formation of cytoplasmic puncta of LC3 [51], and accumulation of the receptor molecule p62 [52]. These methods rely on western blotting or microscopic imaging, which poses challenges in terms of detection sensitivity and quantification. Recently, quantitative analyses using tandem fluorescent tags [53, 54, 55] and halo tags [56] have been developed. However, the experimental system established in this study allows for high‐sensitivity detection and quantification of LC3B using HiBiT activity measurements. This system is expected to provide a novel approach for evaluating autophagy activity from a different perspective than previously available methods.

Compared with the steady state, treatment with BafA1 disrupted lysosomal function, leading to increased intracellular accumulation and extracellular secretion of LC3B (Fig. 3A). This finding is consistent with previously reported analyses of autophagic secretion [34, 57]. Additionally, the detection of LC3B released into the extracellular space was dependent on detergent treatment, indicating that the secretion of LC3B occurs via fusion with the plasma membrane of the autophagosome rather than secretion after transportation to the lysosome. Interestingly, we observed that LC3B released into the extracellular space included not only type II but also type I LC3 (Fig. 5B). This suggests that LC3‐I is secreted in vesicular form.

The mechanisms underlying LC3B secretion are thought to involve five pathways (Fig. 6H). The first pathway is the so‐called autophagy secretion pathway, in which LC3‐II, anchored to the isolation membrane, is encapsulated within the autophagosome and secreted extracellularly. Evidence supporting this pathway includes: (a) the presence of extracellularly encapsulated LC3B (Fig. 2D,E), (b) a decrease in the secretion upon knocking out *ATG5* or *FIP200* (Fig. 5A), and (c) a reduction in the secretion of the LC3BΔG mutant (Fig. 5D). Interestingly, the reduction due to the knockout of *ATG5* or *FIP200* was only observed during BafA1 treatment (Fig. 5A), indicating that this pathway was notably active during the inhibition of lysosomal function.

The second pathway involves secretion of encapsulated LC3B‐II in an autophagy‐independent manner. Even in *FIP200* knockout cells, LC3 is secreted in an encapsulated form (Fig. 5B), which is diminished by the ΔG mutation (Fig. 6E). Recently, an autophagy‐related pathway, known as LC3‐associated phagocytosis (LAP), was reported as the mechanism by which macrophages capture and degrade extracellular pathogens [58, 59, 60], showing that in the LAP, LC3 is anchored to the endosomal membrane in an autophagy‐independent manner. These LC3 molecules may be incorporated into luminal vesicles during multivesicular body formation and secreted extracellularly via the same pathway as in exosomes. Alternatively, LC3 lipidation may occur at the plasma membrane, with the plasma membrane budding to form microvesicles for secretion.

The third pathway involves the secretion of LC3B in association with autophagy receptor molecules. The presence of this pathway was suggested by the decrease in secretion rates during both steady state and BafA1 treatment when using K51A or K51A/L53A mutations, which reduced the binding affinity to LIR (Fig. 6C). Moreover, the absence of a decrease in secretion rates when these mutations were introduced alongside the ΔG mutation (Fig. 6E) implies that primarily nonlipidated LC3‐I may be secreted via this pathway. The decrease in the secretion rate of the ΔG mutant due to *FIP200* knockout (Fig. 6E) may be explained by the inhibition of this pathway.

The fourth pathway involves the secretion of nonlipidated LC3‐I, which is encapsulated and secreted via a different mechanism from that described previously. The LC3B mutant with both ΔG and K51A/L53A mutations is neither lipidated nor capable of binding to the autophagy receptors. This double mutant was confirmed to be secreted in a certain amount, even in *FIP200* knockout cells that exhibited autophagic deficiency (Fig. 6E). Some LC3‐I may be incorporated into the apoptotic bodies and released into the extracellular space.

The fifth pathway involves the secretion of nonencapsulated LC3. LC3B molecules were detected even under nondetergent‐treated conditions in the 100k sup fraction from the centrifuged culture media (Fig. 2E). This may result from the lysosomal secretion that occurs when autolysosomes fuse with the plasma membrane. The fusion of lysosomes may lead to the degradation of the inner double membrane, resulting in the secretion of LC3 molecules separated from the membrane into the extracellular space. As outlined above, this study revealed that LC3B is transported extracellularly via various pathways, rather than via a single route.

The LC3 family is divided into two subfamilies: one consisting of LC3A, LC3B, and LC3C, and the other comprising GABARAP, GABARAP L1, and GABARAPL2 [9, 12, 40]. Although structural differences between the two subfamilies have not been identified, they have been suggested to possess distinct functions [12, 40]. The LC3 subfamily is primarily associated with elongation of the isolation membrane and regulation of autophagosome size, whereas the GABARAP subfamily is implicated in the closure of autophagosomes [12, 61]. In this study, we did not find any significant differences in the extracellular secretions of the two subfamilies, suggesting that these functional differences had minimal impact on secretion.

Analysis of the knock‐in cells established in this study revealed that the amount of LC3B secreted was minimal compared to that of the exosome marker CD63 (Fig. 2H) [22]. Even during the inhibition of lysosomal function by BafA1, the amount of LC3B secreted was considerably lower than that of CD63 (Fig. 2H). This indicates that the frequency of autophagic secretion via fusion with the plasma membrane and autophagosomes was lower than that of exosome secretion resulting from fusion with the plasma membrane and microvesicular bodies. However, the secretion rate of TOMM20 did not differ significantly from that of the LC3B cells. Reports have indicated that mitochondria can also be secreted extracellularly [23]. Mitochondria are representative targets of selective autophagic degradation (mitophagy) [62], and their secretion may occur via mitophagy.

Previous reports have revealed that the fusion of autophagosomes with lysosomes involves a group of SNARE molecules, including syntaxin (STX) 17 and YKT6 on the autophagosome side and VAMP7/8 and STX7 on the lysosomal side [61, 63]. Whether autophagy secretion occurs through the binding of STX17 and YKT6 to another t‐SNARE molecule present in the plasma membrane or whether entirely different unidentified molecules are involved remains to be determined.

Analysis of HiBiT‐LC3B KI cells revealed that the secretion of LC3 into the extracellular space decreased under nutrient starvation (Fig. 5A). This reduction was also observed in the cells treated with BafA1 (Fig. 5A). These results indicate that the culture environment influences autophagy. When cells enter a state of nutrient starvation, they may shut down the autophagic secretion pathway to conserve maximum energy while activating lysosomal degradation. Molecules that function as nutrient sensors include sirtuins [64], AMP‐activated protein kinases [65], and mammalian target of rapamycin [66, 67]. Factors downstream of these nutrient sensor signals may control the transport destinations of autophagosomes [66, 68]. Future analyses using knockout cells for these factors, as well as studies on the effects of inhibitor treatment, are required to elucidate the underlying mechanisms.

In conclusion, analysis using the high‐sensitivity detection system developed in this study for LC3B revealed that LC3B molecules are secreted via various pathways. Additionally, by quantitatively analyzing the changes in secretion levels, we gained insights into the behavior of LC3 within the cells. This system is expected to help elucidate mechanisms underlying autophagy.

## Conflict of interest

The authors declare no conflict of interest.

## Author contributions

KS and EM contributed to conceptualization and writing—original draft. KS, MA, and KM contributed to methodology. KS contributed to investigation. KS and MA contributed to resources. EM contributed to writing—review and editing, supervision, and funding acquisition.

## Data Availability

Data supporting the findings of this study is available from the corresponding author upon request.
